# Comparison of Antimicrobial Activity of *Allium sativum* Cloves from China and Taşköprü, Turkey

**DOI:** 10.1155/2018/9302840

**Published:** 2018-11-27

**Authors:** Ali Yetgin, Kerem Canlı, Ergin Murat Altuner

**Affiliations:** ^1^Izmir Institute of Technology, Department of Biotechnology, Institute of Engineering and Science, Izmir, Turkey; ^2^Dokuz Eylül University, Department of Biology, Faculty of Science, Izmir, Turkey; ^3^Kastamonu University, Department of Biology, Faculty of Science and Arts, Kastamonu, Turkey

## Abstract

In this study, antimicrobial activities of two different samples of *Allium sativum* L. from Turkey (TR) (Taşköprü, Kastamonu, Turkey) and China (CN) were determined. A broad spectrum of Gram-negative and Gram-positive bacteria (17 bacteria) including species of *Bacillus*, *Enterobacter*, *Enterococcus*, *Escherichia*, *Klebsiella*, *Listeria*, *Pseudomonas*, *Salmonella*, and *Staphylococcus* were used for testing antibacterial activity. In addition, antifungal activity against *Candida albicans* was also investigated. Antimicrobial activity was tested by using 3 different processes (chopping, freezing, and slicing by the disk diffusion method). The results showed that TR garlic presented more antimicrobial activity than CN garlic. Mechanism of activity of CN garlic could be proposed to be different from that of TR garlic.

## 1. Introduction

Prevention of food infection, related to pathogens and spoilers, is a significant topic at the last decades [1]. In order to inhibit food pathogens, medicinal plant research supply abundant source as natural preservatives [2]. Garlic is one of the oldest agricultural harvests, which has historical records dating back to BC 800 and today being used worldwide as food and medicine [3]. Garlic has several different uses, such as garlic volatile oil, garlic powder, and garlic juice for its antimicrobial activity. Main garlic species is *A. sativum*, which is not only accepted as an ethnopharmaceutical drug but also proved to have therapeutic effects by several scientific research studies. It has been used as food and medicine starting from ancient times in India, Egypt, Greece, and Rome [4]. Several research studies about antibacterial, antiprotozoal, anticancer, antifungal, and antiviral activity of garlic can be found as the current literature is concerned [5].

The most significant component of garlic is allicin (diallyl thiosulfinate), and its activity is investigated against a broad range of Gram-positive and Gram-negative bacteria. Allicin is not present in fresh clove of garlic, but it is released after crushing and chopping with the alliinase enzyme activity. Alliums, component of garlic, include largely cysteine sulfoxides. Conversion of alliinase to allicin by cysteine sulfoxides transforms to thiosulfates, which are volatile and lachrymatory [6]. Allicin, an organosulfur compound, which prevents lipid biosynthesis, was proved to damage *Candida albicans* cell wall [7] and cause inhibition of RNA synthesis in bacteria [8]. The antimicrobial activities of allicin and garlic extract investigated a large spectrum against *Mycobacterium*, *Photobacterium*, *Proteus*, *Pseudomonas*, *Salmonella*, *Staphylococcus*, *Escherichia*, *Helicobacter*, *Clostridium*, *Cryptocaryon*, *Klebsiella*, and *Bacillus* species [9].

In this study, the antimicrobial activity of *A. sativum* was analyzed after 3 different processes, namely, chopping, freezing, and slicing, by using the disk diffusion method. In addition, the activities of two garlic samples, one from Taşköprü, Kastamonu, Turkey (TR), and the other from China (CN), are also compared.

## 2. Materials and Methods

### 2.1. Garlic

Two kinds of *A. sativum*, from Turkey (Taşköprü, Kastamonu) and China, were obtained for this study from a local company. Garlic, cultivated in Taşköprü region, is free of any chemical treatment; however, garlic cultivated in China region could possibly be treated by chemicals due to its industrial production.

### 2.2. Microbial Strains

Seventeen bacteria and 1 fungus species were used, and these microorganisms were sustained on nutrient agar (BD Difco, USA). There are 11 standard bacteria and 1 standard fungus. Five of them are standard Gram-positive bacteria, which are *Bacillus subtilis* DSMZ 1971, *Enterococcus faecalis* ATCC 29212, *Listeria monocytogenes* ATCC 7644, *Staphylococcus aureus* ATCC 25923, and *Staphylococcus epidermidis* DSMZ 20044. The others are standard Gram-negative bacteria, which are *Enterobacter aerogenes* ATCC 13048, *Escherichia coli* ATCC 25922, *Pseudomonas aeruginosa* DSMZ 50071, *Pseudomonas fluorescens* P1, *Salmonella enteritidis* ATCC 13075, and *Salmonella typhimurium* SL1344. There is 1 standard fungus, which is *Candida albicans* DSMZ 1386. Besides, there are 6 nonstandard bacteria, which are isolated from food at Ankara University microbiology laboratory. Three of them are Gram-positive bacteria, which are *Enterococcus durans*, *Enterecoccus faecium*, and *Listeria innocua*. The others are Gram-negative bacteria, which are *Klebsiella pneumonia*, *Salmonella infantis*, and *Salmonella Kentucky*.

### 2.3. Garlic Ethanol Extracts

Garlic samples were prepared by 3 different processes: chopping, freezing, and slicing. Garlic is chopped in small pieces using a grinder. Garlic is sliced just into two pieces using a knife. In the freezing process, using an ultra freezer, garlic was frozen, and then it was ground immediately using a cold grinder. In all processes, 50 g of garlic was used, and these samples were shaken in ethanol (Sigma-Aldrich) at 125 rpm for 2 days at room temperature [10]. After that, all of them were filtrated using Whatman No. 1 filter paper into evaporation flasks. Filtrates were evaporated by a rotary evaporator (Buchi R3) at 45°C [11]. Finally, remnants were collected, and the quantities used for each process are as given in Table 1. In order to compare the results, the first and second quantities were adjusted to the same values in *µ*g, where the third was set to the same value as *µ*L.

### 2.4. Preparation of Inocula

All bacterial strains were incubated at 37°C for 24 hours; however, *Candida albicans* DSMZ 1386 was incubated at 27°C for 48 hours [12]. Each bacteria and yeast were inoculated into 0.9% sterile saline solution and adjusted to 0.5 McFarland standard, in order to standardize the inocula to contain about 10^8^ cfu·mL^−1^ for bacteria and 10^7^ cfu·mL^−1^ for *Candida albicans* [13].

### 2.5. Antimicrobial Activity Test

The antimicrobial activity of garlic ethanol extract was performed by he disk diffusion test, as described by Andrews [14]. First, Mueller-Hinton Agar (BD Difco, USA) was poured into 90 mm sterile Petri dishes until reaching a mean depth of 4.0 mm ± 0.5 mm. Extracts were loaded on 6 mm Oxoid Antimicrobial Susceptibility Test Disk as given in Table 1. Disks were left to dry overnight at 30°C under sterile conditions in order to prevent any remaining solvent, which may interfere with the result. After that, prepared microorganisms, which were inoculated into the saline solution, were streaked on the surface of Petri dishes. The plates were left to dry for 5 minutes at room temperature under aseptic conditions [15]. Next, disks were tightly applied to the surface of plates. Finally, the plates were incubated, and inhibition zone diameters were observed [16, 17].

### 2.6. Controls

Empty sterile disks and extraction solvent (ethanol) were used as negative controls.

### 2.7. Statistics

The statistical analysis was executed by a nonparametric method, Kruskal–Wallis, which is one-way analysis of variance with *p* < 0.05.

## 3. Results

Antimicrobial activity of *A. sativum* cloves (from Turkey and China) ethanol extracts were analyzed in our study. In order to load extract, empty sterile disks were used, and then these disks were applied on a culture medium (Mueller-Hinton Agar), which is inoculated with microorganisms. Inhibition zones were observed, when the extracts had activity against these microorganisms. The diameter of these zones was measured in millimetres as given in Tables 2 and 3. No activity for empty sterile disks and ethanol loaded on disks and evaporated before application, which are negative controls, was observed.

The diameter of inhibition zones for *A. sativum* from Turkey is given in Table 2. Turkey-chopped garlic (TC) has antimicrobial activity against 17 bacteria; however, Turkey-frozen garlic (TF) has antimicrobial activity against 9 bacteria, whereas Turkey-sliced garlic (TS) has antimicrobial activity against 6 bacteria. According to Table 2, TC has high antimicrobial activity against *B. subtilis* DSMZ 1971 (24 mm), *E. faecium* (17 mm), and *L. monocytogenes* ATCC 7644 (18 mm) at 656.25 mg sample. TF has only high antimicrobial activity against *B. subtilis* DSMZ 1971 (15 mm). Furthermore, TC, TF, and TS have high antifungal activity against *Candida albicans* DSMZ 1385 (30, 19, and 16 mm, respectively); however, the antifungal activity dramatically decreased in TF and TS. These results demonstrate that freezing and slicing negatively affected the antimicrobial activity of *A. sativum* from Turkey.

The diameter of inhibition zones for *A. sativum* from China is given in Table 3. China-chopped garlic (CC) has antimicrobial activity against 15 bacteria, China-frozen garlic (CF) has antimicrobial activity against 15 bacteria, and China-sliced garlic (CS) has antimicrobial activity against 12 bacteria. According to Table 3, CC, CF, and CS have high antimicrobial activity against *B. subtilis* DSMZ 1971 (24, 28, and 23 mm, respectively) and *E. faecium* (17, 18, 14 mm respectively). Furthermore, CC, CF, and CS have high antifungal activity against *Candida albicans* DSMZ 1385 (30, 28, and 30 mm, respectively). These results demonstrate that freezing and slicing did not negatively affect the antimicrobial activity of *A. sativum* from China.

## 4. Discussion

In this research, ethanol is used as an extraction solvent because it has best solvability of active ingredients when compared to other solvents, such as methanol, ethyl acetate, and chloroform [18]. Gram-positive bacteria are more sensitive to antimicrobials, and they have no powerful wall because of the existence of only thick peptidoglycan layers on the outer surface [19]. However, Gram-negative bacteria are less susceptible due to phospholipidic membrane, which prevents the permeability of lipophilic solutes. These hydrophilic solutes can pass with porines, which are selective barriers.

*L. monocytogenes* has critical food-borne pathogen activity, and it causes listeriosis with serious illness [20]. Since the production of ready-to-eat food is increasing, discovering some alternative compounds has become critical against listeriosis for the food industry [21]. For listeriosis treatment, natural garlic product can be used significantly rather than industrial product.

According to Kallel et al. [22], *A. sativum* ethanol extract had moderate antibacterial activity against *B. subtilis* and *S. aureus*, which were 10–15 mm and low-level activity against *B. thuringiensis* and *P. aeruginosa*, which were <10 mm, however, presented no activity against *K. pneumoniae*, *E. coli*, and *S. typhimurium*. Also, Karuppiah and Rajaram [23] reported that *A. sativum* ethanolic extract had antibacterial activity against all tested multiple antibiotic-resistant (MAR) bacteria such as *P. aeruginosa* (19.45 mm), *E. coli* (18.50 mm), *Bacillus* sp. (16.5 mm), *Proteus* sp. (13.50 mm), *Enterobacter* sp. (13.50 mm), and *S. aureus* (13.50 mm).

Our results are also important since a broad range of strains were tested, and as a result, we can propose that TR garlic is more effective than CN garlic. These activity differences are not related to garlic clove amount, because their first and second volumes were equaled at sort of *µ*g, and third volumes were equaled at sort of *µ*L. For this reason, their differences are related to their ingredients.

## 5. Conclusion

According to our results, TR *A. sativum* (Turkey) has more antimicrobial activities than CN *A. sativum* (China). In addition, freezing and slicing negatively affected the antimicrobial activity of *A. sativum* from Turkey, and in contrary, no reverse effect was observed for freezing and slicing against *A. sativum* which is from China. By using freezing and slicing, motor force was prevented in order to inhibit the transformation of alliin to allicin. The process of industrial production could lead to change in antimicrobial activity and composition and concentration of active components. However, further research studies are required in order to analyse these active substances and their mechanism of activity in detail. Besides, these results should be supported by further large-scale studies; however, by keeping in mind that geographical differences can cause active compound differences, these differences must also be taken into account in further studies.

## Figures and Tables

**Table 1:** Amount of garlic samples which were loaded on disks in *µ*g and *µ*L.

Garlic	1.	2.	3.
TC	303.75 *µ*g (112.5 *µ*L)	607.5 *µ*g (225 *µ*L)	656.25 *µ*g (262.5 *µ*L)
TF	303.75 *µ*g (87.5 *µ*L)	607.5 *µ*g (175 *µ*L)	708.75 *µ*g (262.5 *µ*L)
TS	151.9 *µ*g (66 *µ*L)	303.75 *µ*g (131.2 *µ*L)	607.5 *µ*g (262.5 *µ*L)
CC	303.75 *µ*g (51 *µ*L)	607.5 *µ*g (112.5 *µ*L)	1155 *µ*g (262.5 *µ*L)
CF	303.75 *µ*g (51 *µ*L)	607.5 *µ*g (112.5 *µ*L)	1155 *µ*g (262.5 *µ*L)
CS	303.75 *µ*g (87.5 *µ*L)	607.5 *µ*g (175 *µ*L)	708.75 *µ*g (262.5 *µ*L)

**Table 2:** Disk diffusion test result for *A. sativum* from Kastamonu (inhibition zones in mm).

	TC			TF			TS		
	1.	2.	3.	1.	2.	3.	1.	2.	3.
*B. subtilis* DSMZ 1971	17	23	24	10	13	15	-	8	10
*C. albicans* DSMZ 1386	25	30	30	11	16	19	-	9	16
*E. aerogenes* ATCC 13048	7	7	7	-	-	-	-	-	7
*E. durans*	-	8	8	-	-	-	-	-	-
*E. faecalis* ATCC 29212	-	8	9	-	-	7	-	-	-
*E. faecium*	11	17	17	-	10	10	-	-	8
*E. coli* ATCC 25922	-	7	7	-	-	-	-	-	-
*K. pneumonia*	7	7	7	7	7	7	7	8	7
*L. innocua*	7	8	9	-	-	-	-	-	-
*L. monocytogenes* ATCC 7644	18	20	18	-	-	-	-	8	8
*P. aeruginosa* DSMZ 50071	7	7	8	-	-	7	-	-	-
*P. fluorescens* P1	-	-	7	-	-	-	-	-	-
*S. enteritidis* ATCC 13075	-	7	8	-	-	7	-	-	-
*S. infantis*	-	7	7	-	-	-	-	-	-
*S. kentucky*	7	7	7	-	-	-	-	-	7
*S. typhimurium* SL1344	-	-	7	-	-	7	-	-	-
*S. aureus* ATCC 25923	9	11	13	-	7	8	-	-	-
*S. epidermidis* DSMZ 20044	-	9	10	-	7	8	-	-	-

“-”: no activity observed.

**Table 3:** Disk diffusion test result for *A. sativum* from China (inhibition zones in mm).

	CC			CF			CS		
	1.	2.	3.	1.	2.	3.	1.	2.	3.
*B. subtilis* DSMZ 1971	12	21	24	17	19	28	14	20	23
*C. albicans* DSMZ 1386	16	24	30	17	20	28	18	25	30
*E. aerogenes* ATCC 13048	-	7	7	-	-	7	-	-	-
*E. durans*	-	-	8	-	-	8	7	7	7
E. *faecalis* ATCC 29212	-	-	8	-	-	8	-	-	8
*E. faecium*	9	10	17	8	9	18	9	11	14
*E. coli* ATCC 25922	-	7	7	-	7	7	-	7	7
*K. pneumonia*	7	7	7	7	7	7	7	7	7
*L. innocua*	-	-	9	-	-	8	-	-	8
*L. monocytogenes* ATCC 7644	-	8	10	-	9	12	8	9	10
*P. aeruginosa* DSMZ 50071	-	7	7	-	7	7	-	7	7
*P. fluorescens* P1	-	-	9	-	-	8	-	-	-
*S. enteritidis* ATCC 13075	-	-	7	-	-	7	-	-	7
*S. infantis*	-	7	7	-	7	7	-	-	-
*S. kentucky*	-	-	-	-	-	-	-	-	-
*S. typhimurium* SL1344	-	-	-	-	-	-	-	-	-
*S. aureus* ATCC 25923	-	8	10	-	8	11	8	10	11
*S. epidermidis* DSMZ 20044	-	8	9	-	7	8	-	7	9

“-”: no activity observed.

## Abbreviations

CC: China-chopped garlic
CF: China-frozen garlic
CS: China-sliced garlic
CN: Garlic cloves from China
TR: Garlic cloves from Turkey
TC: Turkey-chopped garlic
TF: Turkey-frozen garlic
TS: Turkey-sliced garlic.
